# The effect of staged TIP urethroplasty on proximal hypospadias with severe chordee

**DOI:** 10.3389/fsurg.2022.892048

**Published:** 2022-08-25

**Authors:** Qike Xie, Yuling Liu, Xiangyou Zhao, Junqiang Huang, Chao Chen

**Affiliations:** Department of Pediatric Surgery, The First Affiliated Hospital of Guangxi Medical University, Nanning, China

**Keywords:** hypospadias, penis, urethra, urinary surgery, two-stage surgery

## Abstract

**Background:**

Proximal hypospadias with severe chordee is still a formidable challenge for most pediatric urologists, and the treatment approach remains controversial. Here, we describe a modified two-stage technique to repair proximal hypospadias with severe chordee.

**Methods:**

We retrospectively identified 53 children referred for proximal hypospadias with severe chordee from July 2016 to July 2019, who underwent a two-stage urethroplasty. In group 1, the children were repaired with staged tubularized incised plate (TIP) urethroplasty, while Byars’ two-stage urethroplasty was attempted in group 2. We corrected chordee by releasing all remaining attachments to the corpora after degloving the penis, transceting the urethral plate, and dorsal plication. The mean age of patients in the first stage of surgery was 26.6 months in group 1 and 24.8 months in group 2. Postoperative complications in the two groups included: fistula, urethral stricture, urethral diverticulum, and glanular dehiscence.

**Results:**

A total of 20 cases were repaired with staged TIP urethroplasty (group 1), and 33 cases were repaired with Byars’ two-stage urethroplasty (group 2). The length of follow-up in group 1 was 39.8 ± 10.1 months, and in group 2, it was 38.1 ± 8.7 months (*P* > 0.05). After the second stage of surgery, 1 case (5%) in group 1 and 11 cases (33.3%) in group 2 developed a urinary fistula (*P* < 0.05). One case (5%) in group 1 and three cases (9.1%) in group 2 had urethral stricture (*P* > 0.05). All strictures were cured by repeated dilation, and no patient required reoperation. No cases in group 1 and one case (3%) in group 2 had urethral diverticulum (*P* > 0.05). There was no residual chordee in both groups. Two cases (10%) in group 1 and 13 cases (39.3%) in group 2 required reoperation (*P* < 0.05).

**Conclusions:**

Staged urethroplasty is appropriate to repair proximal hypospadias with severe chordee. Particularly, staged TIP urethroplasty is a good choice for patients with proximal hypospadias and severe chordee, especially those with better penile development, wider urethral plate, larger glans, and deeper navicular fossa of the urethra.

## Introduction

Hypospadias is the most common deformity of the male genitourinary system, but its precise etiology is still undefined. Baskin reported that the incidence of male hypospadias was 1/300, and proximal hypospadias accounted for 20% (1). The urethral plate of the proximal hypospadias is usually unclear, short, fibrotic, and stretched, resulting in a high incidence of severe chordee. The foreskin of the proximal hypospadias is asymmetrical and accumulates on the dorsal side of the penis, while the ventral foreskin is short of traction (2). The repair of proximal hypospadias with chordee is still a formidable challenge (3–5). Currently, two-stage operations have become the standard of care (6–8); however, selection between flaps and grafts has been and remains a matter of controversy.

We describe a new modified technique for staged hypospadias repair. In patients with good penile development, wide urethral plate, larger glans, and deep navicular fossa of the urethra, we applied tubularized incised plate (TIP) urethroplasty to repair the distal portion of the urethral defect in the first stage. All patients were applied the Thiersch–Duplay procedure to repair the residual urethral defect during the second-stage operation. The overall success rate was 91.5%–96.6% in the tubularized incised plate urethroplasty (9, 10), while it was 53%–88.2% in Byars’ two-stage repair (2, 11–13). In our research, the overall success rate of staged TIP urethroplasty (90%) is higher than that of Byars’ two-stage repair (60.6%). Staged TIP urethroplasty has a lower incidence of postoperative complications and better clinical effect. Therefore, TIP-staged urethroplasty should be considered a choice for patients with proximal hypospadias and severe chordee, especially those with better penile development, wider urethral plate, larger glans, and deeper navicular fossa of the urethra.

## Materials and methods

### Study design

We retrospectively identified 53 children with proximal hypospadias with severe chordee from July 2016 to July 2019. All of them were repaired with staged urethroplasty. Twenty children underwent staged TIP urethroplasty (group 1), and 33 were managed by Byars’ two-stage urethroplasty (group 2). According to the position of the ectopic urethral orifice, hypospadias was divided into proximal penile and penoscrotal. Glans diameter was measured at the point of the maximum glans width. These patients had a karyotype of 46,XY, and a penile curvature of more than 45°. The choice of surgical technique depended on the development of the penis.

### Surgical technique

**Figure 1:**
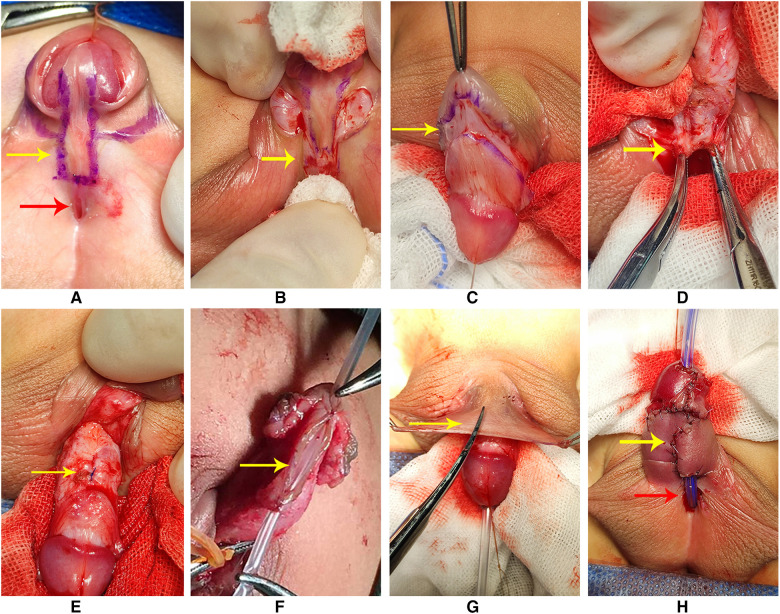
(**A**) Designing of the incisions (red arrow: the ectopic urethral orifice). (**B**) Transection of the urethral plate (arrow); a longitudinal parallel incision with a width of about 8–10 mm. (**C**) Degloving the penis (arrow). (**D**) Mobilizing the urethral plate along the base of the urethra cavernosum (arrow: proximal urethral plate). (**E**) Dorsal plication was made to straighten the penis (arrow). (**F**) Urethral plate was rolled into a tube with a running suture to form the neourethra. (**G**) Mobilizing and transferring the flap to the ventral of the penis to cover the neourethra and corpus cavernosum (arrow). (**H**) Completing the urethroplasty (red arrow: residual urethral defect and the ectopic urethral orifice; yellow arrow: neourethra).

In group 1, we designed the incisions (Figure 1A). Then, we transected the urethral plate 5 mm above the ectopic urethral orifice and made a longitudinal parallel incision with a width of about 8–10 mm along both sides from the glans to the transected urethral plate (Figure 1B). A circumferential incision proximal to the corona was made to deglove the penis subsequently (Figure 1C). When transecting the urethral plate was completed, the urethral plate was mobilized along the base of the urethra cavernosum (Figure 1D). After degloving the penis, transceting the urethral plate, and releasing all remaining attachments to the corpora completely, we performed the artificial erection experiment to assess the penile curvature. If there was residual chordee in the penis, we used a dorsal tunica plication technique at the point of the maximum curvature to straighten the penis (Figure 1E). We dissected and separated the wings of the glans. Indwelling a 6F silicone catheter, the urethral plate was rolled into a tube with a running suture to form the neourethra (Figure 1F). The neourethra and the ectopic urethral orifice were respectively sutured to the tunica albuginea of the corpus cavernosum. We mobilized and transferred the fascial pedicle flap from the dorsal prepuce to cover the neourethra and the corpus cavernosum (Figure 1G). This completed the urethroplasty procedure (Figure 1H). The 6-zero absorbable monofilament sutures were applied for all layers.

In group 2, we made a circumferential incision that was proximal to the corona, which reached the depth of the Buck fascia, allowing for degloving and chordee release. After degloving the penis, transceting the urethral plate, and releasing all remaining attachments to the corpora completely, we performed an artificial erection experiment to assess the penile curvature. If there was residual chordee in the penis, then we applied the dorsal tunica plication technique at the point of the maximum curvature to straighten the penis. Then, the glans were split to facilitate dissection of the glans wings. The dorsal foreskin was unfolded and divided at the midline. The most distal portion of the inner prepuce was rotated into the glanular cleft and sutured to the mucosa of the glans. A midline closure was then performed with the Byars flap. The urethral catheter was removed 7–10 days postoperatively.

All patients received intravenous antibiotics 3–5 days postoperatively. Six months later, a second-stage operation was performed to repair the residual defect of the urethra. The Thiersch–Duplay procedure was applied to all patients to repair the urethral defect during the second-stage operation.

**Figure 2:**
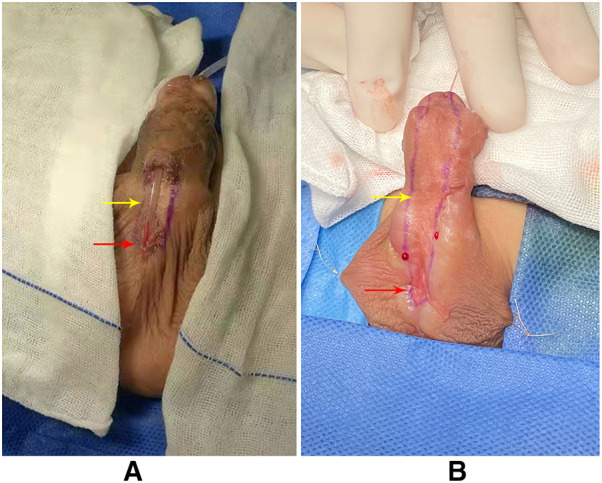
(**A**) Appearance 6 months after the first stage of TIP urethroplasty (red arrow: the ectopic urethral orifice; yellow arrow: residual urethral defect). (**B**) Appearance 6 months after the first stage of Byars’ two-stage urethroplasty (red arrow: ectopic urethral orifice; yellow arrow: residual urethral defect).

**Figure 3:**
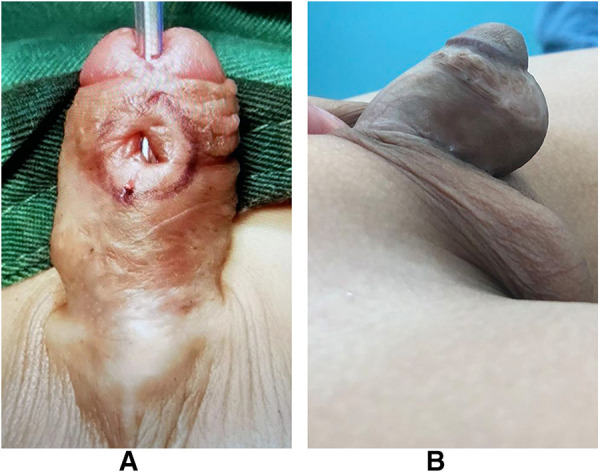
(**A**) Fistula. (**B**) Diverticulum.

**Figure 4:**
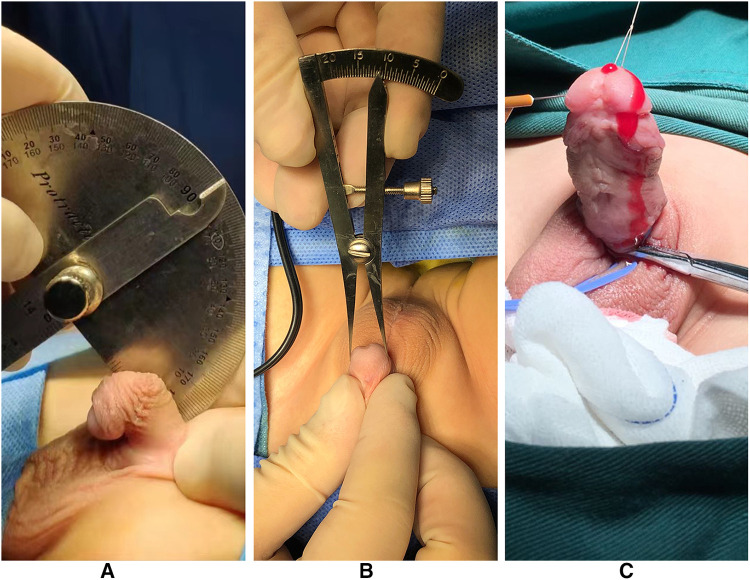
(**A**) Measuring the penile curvature. (**B**) Measuring the glans diameter. (**C**) Artificial erection experiment at the second stage of operation.

Figure 2 was the appearance 6 months after the first stage of urethroplasty. Figure 3 was the complications of urethroplasty. Preoperative measurement was seen in Figure 4.

### Follow-up

All patients attended our patient assessments at 1, 3, 6, and 12 months after completion of the second stage of surgery. Then, we planned follow-up visits once a year. During the follow-up period, the appearance of voiding and uroflowmetry were evaluated.

### Statistical analysis

Differences between the two groups were analyzed using the chi-square test and the *t*-test. Data were expressed as mean ± SD, with statistical significance considered at *P* < 0.05. All analyses were performed using IBM SPSS Statistics software (version 21).

## Results

The age at the first stage of surgery ranged from 6.5 to 78 months, and there were no significant differences between the groups (Table 1). In total, 17 patients had the ectopic urethral orifice located on the proximal penile, while 3 patients had the ectopic urethral orifice located on the penoscrotal in group 1. In group 2, 25 patients had the ectopic urethral orifice located on the proximal penis, while 8 patients had the ectopic urethral orifice located on the penoscrotal. There was no significant difference in the distribution of urethral meatus between the groups. The glans diameter was more than or equal to 12 mm in group 1, while it was more than or equal to 12 mm in 9 patients and less than 12 mm in 24 patients in group 2. After the penis was straightened, the mean ± SD length of the urethral defect was 4.88 ± 1.18 cm in group 1 and 5.15 ± 1.23 cm in group 2 (*P* > 0.05). After the first stage of operation, the length of the residual urethral defect was 2.32 ± 0.58 cm in group 1 and 5.15 ± 1.23 cm in group 2 (*P* < 0.05). The length of follow-up in group 1 was 39.8 ± 10.1 months and 38.1 ± 8.7 months in group 2 (*P* > 0.05).

**Table 1:** Characteristics of patients.

	Group 1	Group 2	*p*-Value
Total patients	20	33	
Age at urethroplasty months, Md (min, max)	26.6 (6.5–78)	24.8 (10–70)	>0.05
Glans diameter (mm)			<0.05
Less than 12	0	24	
More than or equal to 12	20	9	
Hypospadias location			>0.05
Proximal penile	17	25	
Penoscrotal	3	8	
Urethral defect after penis extension (cm)	4.88 ± 1.18	5.15 ± 1.23	>0.05
Residual urethral defect (cm)	2.32 ± 0.58	5.15 ± 1.23	<0.05
Length of follow-up (months)	39.8 ± 10.1	38.1 ± 8.7	>0.05

The complications included fistula, stricture, glanular dehiscence, and urethral diverticulum (Table 2). One case (5%) in group 1 and 11 cases (33.3%) in group 2 developed a urethral fistula (*P* < 0.05). One case (5%) in group 1 and three cases (9.1%) in group 2 had urethral stricture (*P* > 0.05). No cases in group 1 and one case (3%) in group 2 had urethral diverticulum (*P* > 0.05). One case (5%) had glanular dehiscence in group 1 and one case (3%) had this condition in group 2. There was no residual chordee in both groups. Two cases (10%) in group 1 and 13 cases (39.3%) in group 2 required reoperation (*P* < 0.05). The patients who required reoperation in group 1 included one patient who suffered fistulas and another who suffered glanular dehiscence. The patients who required reoperation in group 2 included 11 patients who suffered fistulas, 1 who had diverticulum, and another who suffered glanular dehiscence. They had to be reoperated because of the penile appearance and urination. No urethral strictures required reoperation after multiple dilations. None of the patients of the two groups had residual chordee.

**Table 2:** Surgical complications.

	Group1	Group2	Difference^a^	*P*-value
Total patients	20	33		
Fistulas	1	11	4.205	<0.05
Strictures	1	3	0.000	>0.05
Diverticulum	0	1	0.623	>0.05
Glanular dehiscence	1	1	0.617	>0.05
Reoperation	2	13	5.302	<0.05

^a^ Based on the chi-square test and Fisher's exact test.

## Discussion

In 1994, Snodgrass officially reported TIP surgery for distal hypospadias (14). In 2009, Snodgrass reported that mobilization of the corpus spongiosum/urethral plate and the urethra in proximal hypospadias cases with greater than 30° ventral curvature after penile degloving reduces the need for urethral plate transection (15).

In recent years, research on staged surgery for patients with severe hypospadias has regained people's hopes. Generally, staged surgery is used to increase the repair materials available for the second stage of surgery—foreskin, glans circumference, and tissue blood vessels.

Although staged surgery is more time-consuming and more expensive than others, it usually provides a healthier urethral plate and reduces scar tissue around the urethra (16). A review of the latest literature shows that most patients repaired by staged surgery have excellent cosmetic and functional results (4, 15). Besides, Shukla et al. reported cases of 700 patients with proximal hypospadias who underwent staging operations, which reduced the incidence of urinary fistulas, ruptured glands, and urethral strictures, and achieved satisfactory appearance (7). Furthermore, Castagnetti and El-Ghoneimi recently published a systematic review of 20 years of publications on severe primary hypospadias management; lower complication rates were shown with a staged approach (17).

TIP urethroplasty formed a centrally and vertically positioned meatus that resembled the normal urethral meatus; in addition, it is thought to allow for better cosmetic results (18). On the other hand, it has been shown that the incision of the urethral plate heals with epithelialization without extensive scar tissue. Studies have shown that TIP is beneficial in improving urine flow rate after surgery. Al Adl et al. reported that TIP improved the maximum urine flow rate 36 months postoperatively (19). Also, Andersson et al. reported that there is great potential for the normalization of urinary flow at puberty for boys treated for hypospadias with TIP urethroplasty (20). In addition, Tijani et al. reported that TIP urethroplasty in a two-stage flap urethroplasty for proximal hypospadias appears to prevent the development of diverticulum (21). Therefore, we applied staged TIP urethroplasty to repair proximal hypospadias with severe chordee.

In the present study, the glans diameter was greater than 12 mm in group 1, and we traversed the urethral plate, released all remaining attachments to the corpora after degloving the penis, and made a dorsal plication to straighten the penis. After the penis was straightened, the mean ± SD length of the urethral defect was 4.88 ± 1.18 cm in group 1 and 5.15 ± 1.23 cm in group 2. As the length of the defect was too long, we did not have enough flap to repair it. Therefore, we chose the two-stage repair for both groups in order to reduce the rate of complication.

In our study, two-stage urethroplasty used the fascial pedicle flap from the dorsal prepuce in the first stage. On the one hand, it completed a part of the urethra creation; on the other hand, it prepared the urethral plate for the second stage of operation. The residual urethral defect in group 1 was 2.32 ± 0.58 cm and 5.15 ± 1.23 cm in group 2 (*P* < 0.05), which meant the staged TIP urethroplasty reduced the length of the urethral defect and decreased the risks of complication at the second stage of surgery.

One case (5%) in group 1 and three cases (9.1%) in group 2 had a urethral stricture in the present study. No patients required reoperation; all strictures were cured by multiple dilations. According to our experience, strictures were commonly caused by a circular anastomosis between the neourethra and the native urethra. The Thiersch–Duplay procedure involved tubularization of the urethral plate following two parallel longitudinal incisions around the edges of the plate; this method was widely used in the second stage of hypospadias repair (22, 23).

There are a few researchers who applied staged TIP urethroplasty to repair proximal hypospadias with severe chordee. Compared with Byars’ two-stage urethroplasty, Arshad (12) and McNamara et al. (24) reported 18% and 29% fistula rates, while a fistula rate of 5% was reported in the staged TIP urethroplasty in our study. McNamara et al. (24), Yang et al. (11), and Wani et al. (2) reported 3%, 3.9%, and 4.4% of glans dehiscence with Byars’ two-stage repair, while one patient (5%) suffered glans dehiscence in our research. The incidence of diverticula formation was seen in 2% of patients, which Wani et al. (2) reported in their research. In our study, no patient had diverticula formation and underwent staged TIP urethroplasty. From the present study, the overall success rate of staged TIP urethroplasty (90%) was higher than that of Byars’ two-stage repair (60.6%). Staged TIP urethroplasty had a lower complication rate and a better clinical effect.

The limitations of the present study include an insufficient number of cases and the unavailability of long-term follow-ups. The lack of preoperative and postoperative uroflowmetry is considered another limitation, which requires further research.

## Conclusion

In this report, staged TIP urethroplasty had a lower complication rate compared with Byars’ two-stage urethroplasty, and it is particularly appropriate for repairing proximal hypospadias with severe chordee.

## Data Availability

The original contributions presented in the study are included in the article/Supplementary Material; further inquiries can be directed to the corresponding author.
